# Effects of thermal treatment of food using barbecue fuels on ambient air and beach sands within recreation facilities

**DOI:** 10.1038/s41598-023-45023-4

**Published:** 2023-10-17

**Authors:** Sebastian Kuś, Iwona Jelonek, Zbigniew Jelonek

**Affiliations:** Faculty of Natural Sciences, University of Silesia in Katowice, 12 Bankowa Street, 40-007 Katowice, Poland

**Keywords:** Ecology, Environmental sciences

## Abstract

Organised bathing areas located within leisure facilities, in addition to the function typical of their purpose, allow in most cases the use of their own barbecue facilities. This type of cooking of food before consumption is very popular during leisure time at the waterfront. However, cooking food on a barbecue produces fumes emitted from both the fuel being burned and the food being grilled. In addition, the fat from the grilled food falls on the hearth, contributing to the release of further toxic compounds and, in many cases, together with other exhaust fumes, causing considerable smoke and the summer smog effect. Such cumulative gases emitted by irregularly dispersed barbecues repeatedly irritate the respiratory tract of beachgoers in the area of these devices, and the residue of unburned barbecue fuel contaminates the resting area. Small pieces of charcoal of various textures tend to sink into the sand when exposed to the elements and can pose a risk to young children playing on the beach by causing choking and minor injuries. The study revealed an assumed range of exposure to dust and gases emitted from barbecuing that extended up to 40 m from the hearth. Additionally, it was demonstrated that the thermal processing of food using barbecue fuels could lead to increased contamination of beach sands from the fuel itself and food storage materials. Therefore, taking into account the studies carried out showing the adverse effects of active barbecues on beach sands and, above all, the atmospheric air and directly on beachgoers, administrators of recreational facilities should strive to concentrate these devices at a distance (up to several tens of metres) from beaches and bathing areas.

## Introduction

Recreation is inseparably linked to the concept of active use of leisure time, a period of relaxation in the modern sense that emerged at the turn of the twentieth century. Spending leisure time at a leisure facility such as a swimming bath in its conception should lead to physical and mental regeneration of the people resting there^1^. While a friendly and aesthetically pleasing environment is necessary for mental balance, cleanliness of the ground, water and air is essential for rebuilding vitality in addition to physical activity. The air we breathe is probably the most important factor from which it is difficult for us to isolate ourselves if it is contaminated^2^. We can limit our contact with the water at the bathing area by using swimming equipment, and we can effectively separate ourselves from the sand with footwear and deckchairs. On the other hand, the filtering of atmospheric air would be extremely cumbersome with the use and installation of appropriate filters^3^. Therefore, taking care to maintain high air standards in terms of above-normal air pollutants in the form of dust PM2.5, PM10^4^ and chemical compounds (H_2_S, NH_3_, Cl_2_, HCHO, SO_2_, CO, NO_2_, RI*) at public facilities is very important. Eliminating or consciously targeting the transmission of air pollutants generated during barbecuing processes in charcoal and charcoal briquette-fuelled barbecuing equipment^5^ is an important element in the management of recreational facilities. It should be mentioned that apart from the mere noticeable visual effect in the form of smoke and the intense smell of burning fuel combined with the smell of fried food altogether causing discomfort to beach users, uncontrolled dispersed barbecuing carries many other hazards^6^. The most annoying and perceptible are the irritants (RI) emitted during the heat treatment of food, which, in addition to the mere effect of odour discomfort, also carry adverse effects on the entire human body^7^. By irritating the respiratory tract, they can cause inflammation and allergic reactions and contribute to the formation of long-term rhinitis in the respiratory system^8^. As a result of prolonged exposure and inhalation of the lungs, nose, mouth, larynx and trachea, the conditions caused by these substances also contribute to the formation of carcinogenic reactions in these organs^9^. Another hazardous component of barbecue smoke is particulate matter, and the spectrum of this emission includes most carcinogenic dust characterized by a diameter between 2.5 and 10 microns^10^. The chemical compounds formed under the influence of high temperatures in reaction with nitrogen, especially nitrogen oxides in higher concentrations, can adversely affect the entire human body as well as human mental health^11^. High concentrations of the abovementioned oxides may accumulate in living organisms both for a short period or in relation to staying in this type of polluted atmosphere with low nitrogen oxide concentration, but for a longer time^12^. A highly dangerous gas that poses a health risk when inhaled is also carbon monoxide. While carbon dioxide in the open air may have a slightly suffocating effect, inhalation of carbon monoxide manifests itself through headaches and nausea. It should be emphasized that the duration of inhalation in the open air is of great importance in the case of CO inhalation, while the concentration of the gas is lower when barbecuing^13^. The other gases isolated during the study, i.e. H_2_S, Cl_2_, HCHO, and SO_2_ may also cause adverse health effects due to their prolonged inhalation. These include inflammation of the respiratory tract and mucosal irritation leading to erosions and tissue degeneration^14^. The authors did not perform a measurement analysis of the VOCs and PAHs emitted in the barbecue exhaust due to the static measurement time needed to record them. Nevertheless, it is essential to highlight that these components of barbecue exhaust are equally detrimental to human health as the other gases analyzed in the experiment above^15,16^. In the vicinity of active barbecue facilities, less active beachgoers tend to be the most vulnerable to inhaling fumes. Preferring a leisure pattern of lounging and sunbathing results in constant exposure to fumes emitted during food preparation from both burnt fuels and grilled food. A different topic, although closely related to the use of barbecue fuels and equipment, is the maintenance of clean beach sands. In most cases, many solid (unburned) elements in the form of charcoal, aluminum foil and organic residues remain in the barbecue ashes after the barbecue is over. Wood charcoal elements of various sizes, shapes, and forms enter and mix with the beach sand after the BBQ process.The visual consequence of this is that the sand turns from a light yellow colour to a less aesthetically pleasing grey colour and poses a potential health risk to beachgoers by causing possible injuries and infections. This risk is compounded by the amount of additional unwanted solid contaminants in the beach sands associated with both the barbecue process and the presence of significant numbers of people in a small area^17^. The main problem in calculating the surface of the beach in terms of charcoal and litter in the sands by visual inspection is their construction. Sandy beaches are made or naturally filled with loose sedimentary rocks, which contributes to solid elements sinking into their structure over time. The movement of the sand itself, caused by natural factors^18^ (precipitation, wind) as well as the mixing of the substrate by beach users themselves through direct physical contact, contributes to this.

$${\text{RI}}* \, - {\text{ O}}_{{3}} ,{\text{ HC}}{.}$$

## Research area

**Figure 1:**
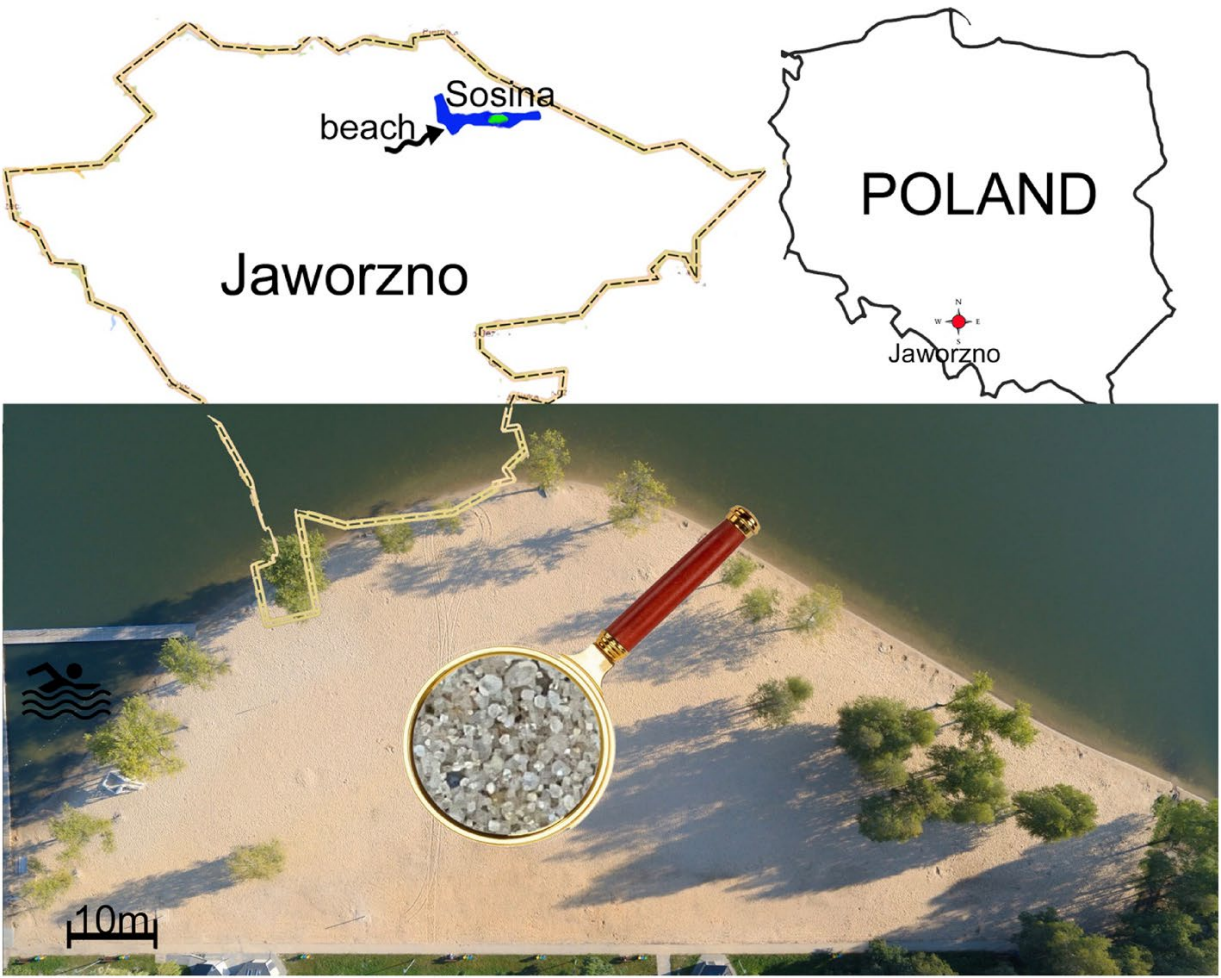
Location of the study area (beach at the "Sosina" reservoir).

A study of the effects of the exhaust fumes from barbecue burners on air composition was carried out at the recreation and sports centre, "Sosina". The site is located approximately 10 km from the centre of the Polish city of Jaworzno in central Europe (50°14′23.1"N 19°19′41.3"E). The recreational reservoir in question is a relatively young reservoir created on the site of the former "Szczakowa" Foundation Sand Mine. The pit was filled with water after mining had ceased and, thanks to the sandy substrate, was quickly adapted for recreation by the local population. The shallow expanse of the "Sosina" reservoir is also particularly conducive to the use of the beach, which is covered with pale yellow sands with grain sizes from 0.1 to 2 mm (Fig. 1).

**Figure 2:**
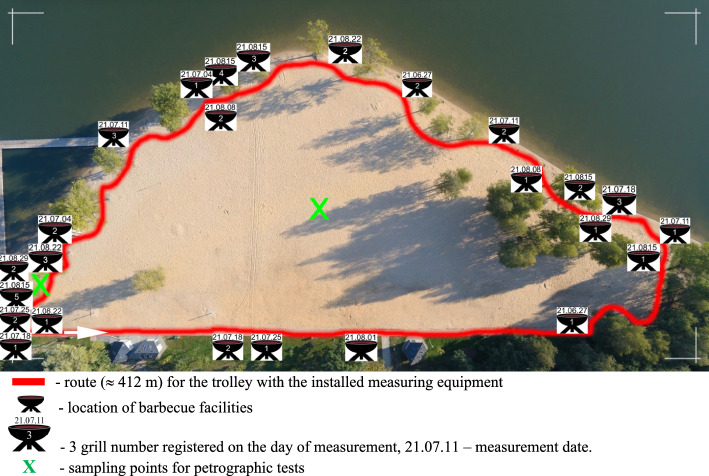
The trajectory of the drive with the measuring apparatus along the beach.

The all-day use of the recreation area is conducive to the decision to prepare one's own meals, and as the resort's regulations do not prohibit barbecues, this method has been successfully adopted in the area. The semicircular beach slopes gently down to the floodplain to the north and is bounded on the south by an escarpment with sparse trees. Barbecue pitches are not located in the centre of the beach for natural reasons (wind in the open). Usually, the barbecues are located along the shoreline bordering the water or on the north side of the beach, where the barbecues are protected from the wind by a natural escarpment and vegetation (Fig. 2).

## Methodology

### Measuring emissions from barbecues

To determine the impact of food processing using barbecue grills with charcoal and charcoal briquettes on the ambient air, tests were carried out in the vicinity of the active appliances. Two Atmon FL^19^ analyzers, certified in accordance with PN-EN standards^20,21^ and equipped with 10 measuring sensors, were used to measure individual gases and aerosols contained in the air in the study area. The concentrations of individual gases were determined in the air under study: CO, RI, HCHO, NH_3_, H_2_S, SO_2_, NO_2_, Cl_2_ and particulate matter PM2.5 and PM10, included in the smoke emitted from barbecue equipment. Air temperature and humidity, atmospheric pressure, precipitation totals, wind direction and speed, and the percentage of total cloud cover during each measurement were also recorded. The tests were performed in 2021 during the bathing season on every Sunday of the month starting from 27 June to 26 September. A total of 11 measurement cycles were carried out, covering the same driving trajectory in the vicinity of active barbecue fireplaces. The measuring apparatus was mounted on a wheeled trolley, allowing it to move along a fixed measurement trajectory of approximately 412 m (Fig. 2). It took approximately 8 min (at an average speed of 3 km/h) for the entire trajectory to be covered, during which measurements were taken by the Atmon FL analysers at one-second intervals.

Barbecues were driven past at a constant speed between 0.5 m and 0.7 m. Because the barbecue equipment was most often run between 1:00 PM and 3:00 PM, measurements of the exhaust fumes emitted by the barbecue equipment were carried out during this time interval.

### Determination of toxic element content

**Figure 3:**
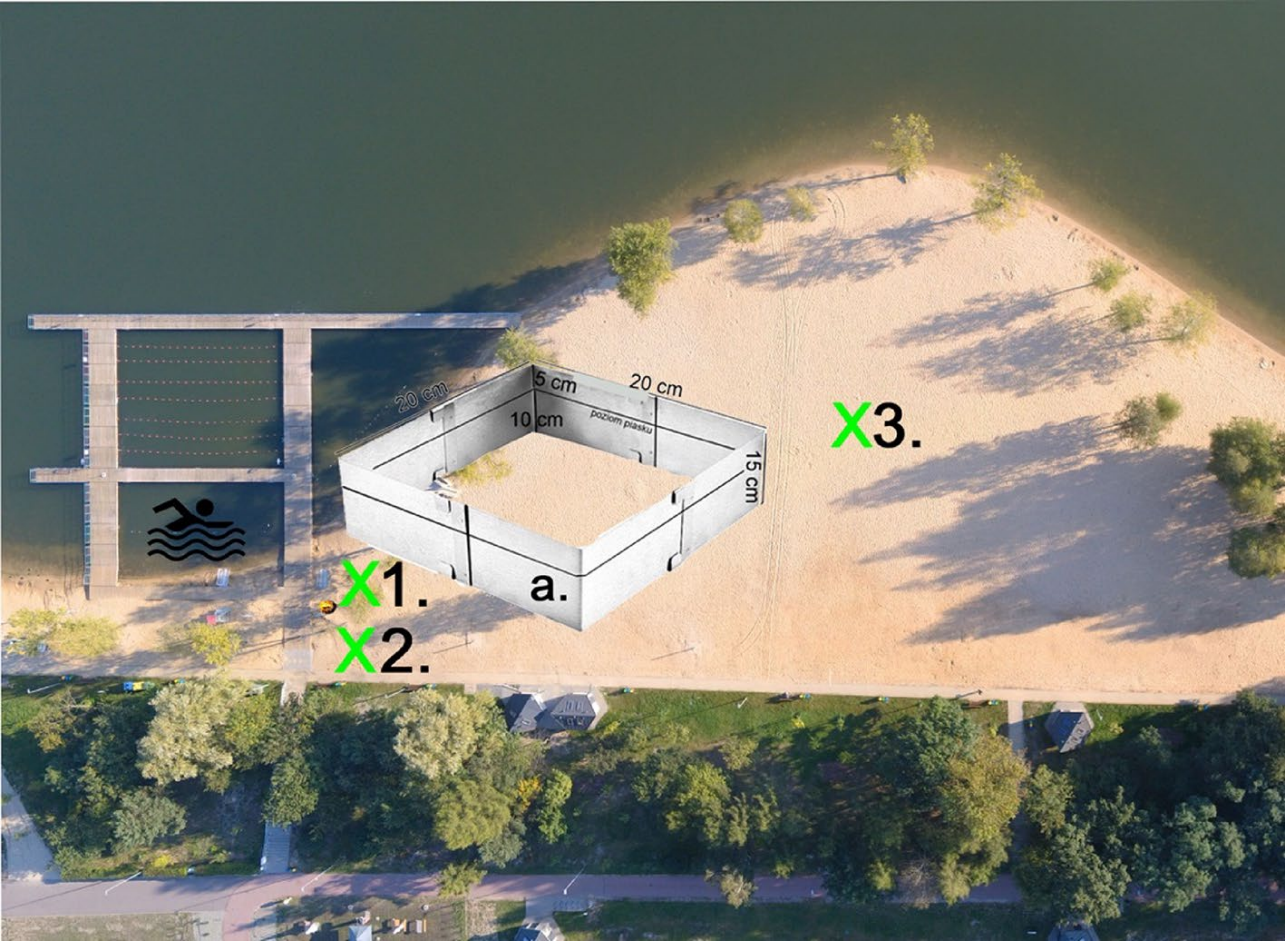
Location of beach sand sampling, a former for sampling loose sedimentary rocks.

To determine the impact of barbecuing on the quality of beach sands, the sand was tested for twelve elements considered toxic. The concentrations of each element in the test material were also determined, and the results were compared with reference values. The sands were analysed in situ in the area of the jetties marking the bathing area (50°14′22.8 "N 19°19′37.6 "E—Fig. 2). This area was selected because it had the highest concentration of barbecue equipment. Measurements were taken at a distance of approx. 5 m (measurement no. 1) and 9 m (measurement no. 2) from piers built on the beach at the "Sosina" reservoir. Figure 3. One measurement (measurement no. 3) was also taken at a site where no active barbecue facilities were found and which was 30 m away from the nearest identified barbecues during the entire study period. The beach sands were analysed in situ using a Vanta M-series instrument (X-ray fluorescence analyser)^22^ measuring the concentrations of individual elements (As, Ba, Cr, Sn, Zn, Cd, Co, Cu, Mo, Ni, Pb, Hg).

### Determination of the components of solids in beach sands

Three samples of beach sands were also taken to determine the qualitative and quantitative impact on beach pollution of solid elements such as fossil^23,24^ and charcoal, coke, slag, glass, metals, biomass, rust, and petroleum products (plastics, oils, grease, etc)^25^. Analogous to the analysis for toxic elements, sands were taken from the area of the highest accumulation of barbecue equipment and sequentially each sample separately from points 1, 2, and 3 (Fig. 3). The sands were sampled using a 20 cm × 20 cm × 15 cm mold, obtaining approximately 600 g of test material each time. The material thus collected was ground with a grinder to a grain size of approximately 2 mm. Subsequently, each sample was passed through a set of sieves 1.5 mm–1 mm–0.5 mm, and from the 0.5 mm sieve, random material (approximately 15 g) was taken with a measuring cup. The collected material was placed in a 40 mm diameter mold and cold-included in Struers' SpeciFix-20 Kit resin. Once the resin had set, the specimen was sanded using 400- and 1200-grit aqueous paper, removing excess resin and levelling the test surface of the specimen. The specimens prepared in this way were subjected to white-light microscopic observation in oil immersion after rinsing with an ultrasonic bath. A ZEISS AxioImager M2m optical microscope with an automatic 75 × 50 scanning table and MCW-2 ECO control panel was used to observe and determine the qualitative and quantitative composition using the oil immersion method at 500 × magnification. For each sample, 1000 measurements were taken during the automatic slide, counting each identified component separately at the intersection of the crosshairs placed in the binocular, and the volumetric composition was automatically calculated in the analysis software^26^.

## Results and discussion

During test runs around the beach (Fig. 2) with the measuring apparatus, significant or smaller variations in measurement readings were found when approaching and moving away from active barbecues. Factors determining weather conditions were also included in the analysis of the extent of the impact of barbecue fumes (Table 1). Mainly wind direction and wind strength were included in the study as the factors most influencing the dispersion or concentration of the measured fumes. The other weather indications, i.e. The air temperature, level of total cloud cover and precipitation did not significantly affect the smoke area but probably had a strong influence on the number of active barbecue devices on a given measurement day.

**Table 1:** Summary of weather parameters on each study day*.

Date	Hour [PM]	Temperature [C]	Precipitation total [mm]	Humidity [%]	Wind speed [km/h]	Wind speed—from	Cloud cover total [%]	Pressure [hPa]	Active grills
27.06.2021	02:00	24.7	0	60.1	3.6	W	41	987	3
04.07.2021	01:45	24.1	0	44	11.9	WNW	35	982	2
11.07.2021	01:30	24.8	0	59.5	18.7	ENE	94	986	3
18.07.2021	01:50	27.8	0	66.6	5.5	N	67	984	3
25.07.2021	01:30	23.3	0.1	49.9	12.2	S	55	982	2
01.08.2021	01:40	21.2	0.8	80	7.7	SEZ	60	976	1
08.08.2021	01:55	23	0	57.5	6.1	WSW	14	983	2
15.08.2021	02:05	28.9	0	48.5	1.4	W	26	984	5
22.08.2021	01:35	25.2	0	45.8	2.8	S	67	978	3
29.08.2021	01:30	22.4	0.1	78.7	5	NNE	14	982	2
05.09.2021	01:30	18.2	0	56.6	22.7	E	0	994	0

*Weather data was taken from the weather station database Davis Vantage Pro2 ,,Sosina” (https://www.weatherlink.com/embeddablePage/show/8137febbe3d74699b2ebcb21c31b1d90/wide).

**Figure 4:**
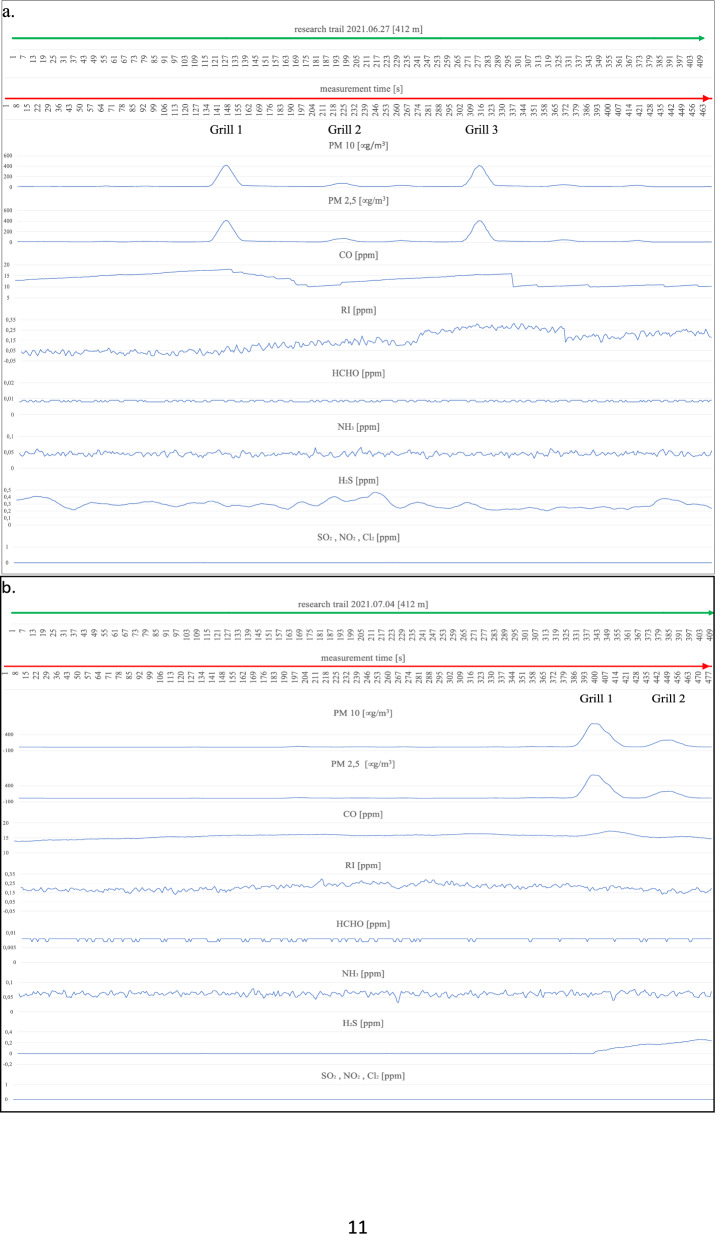

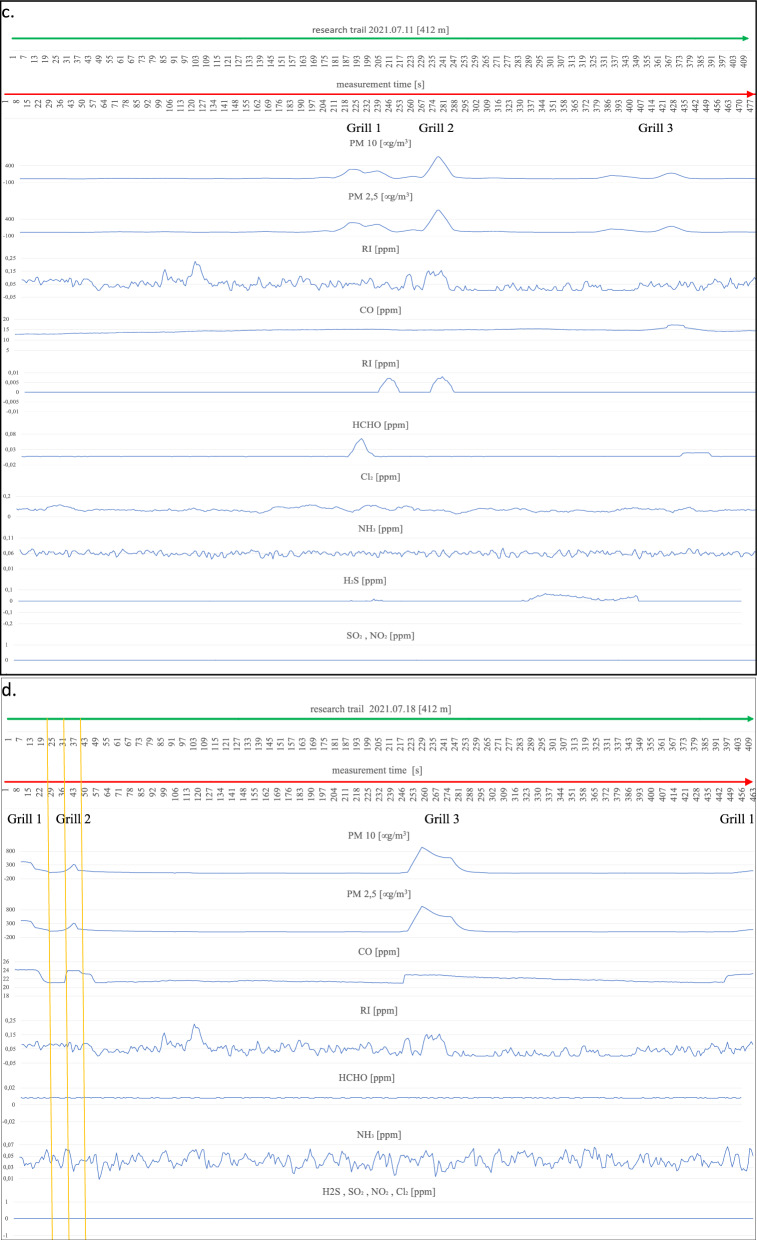

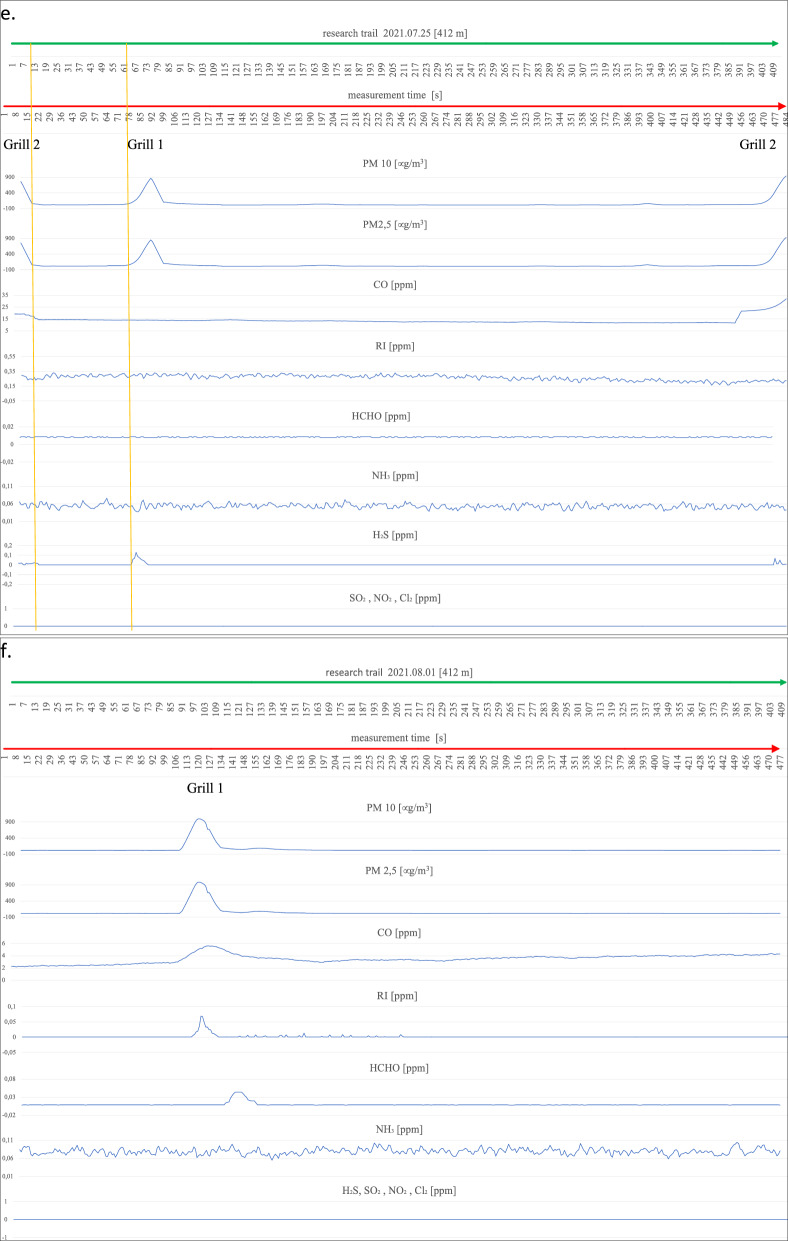

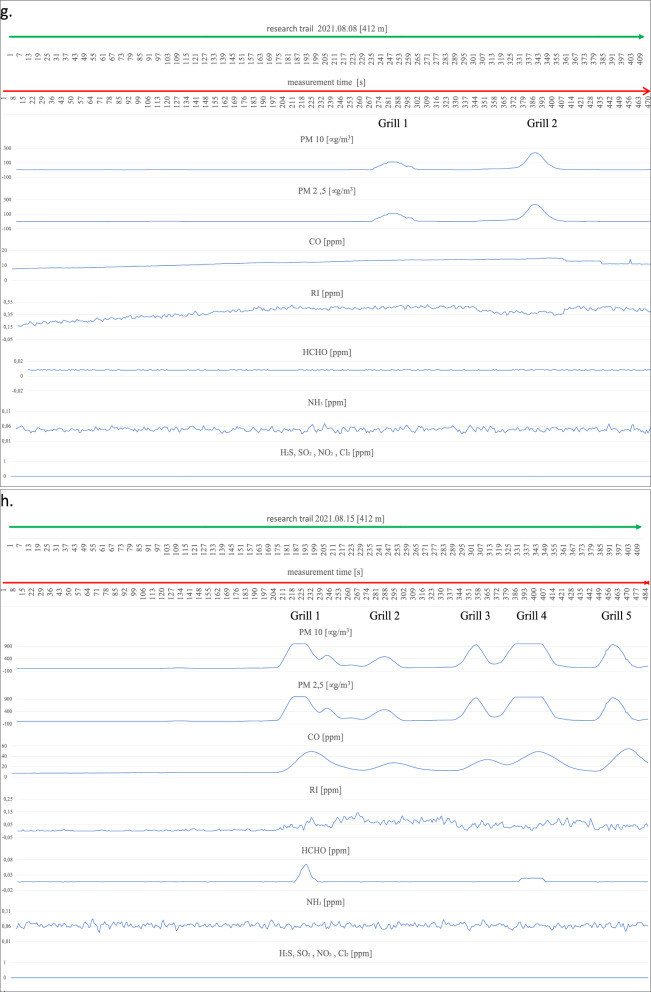

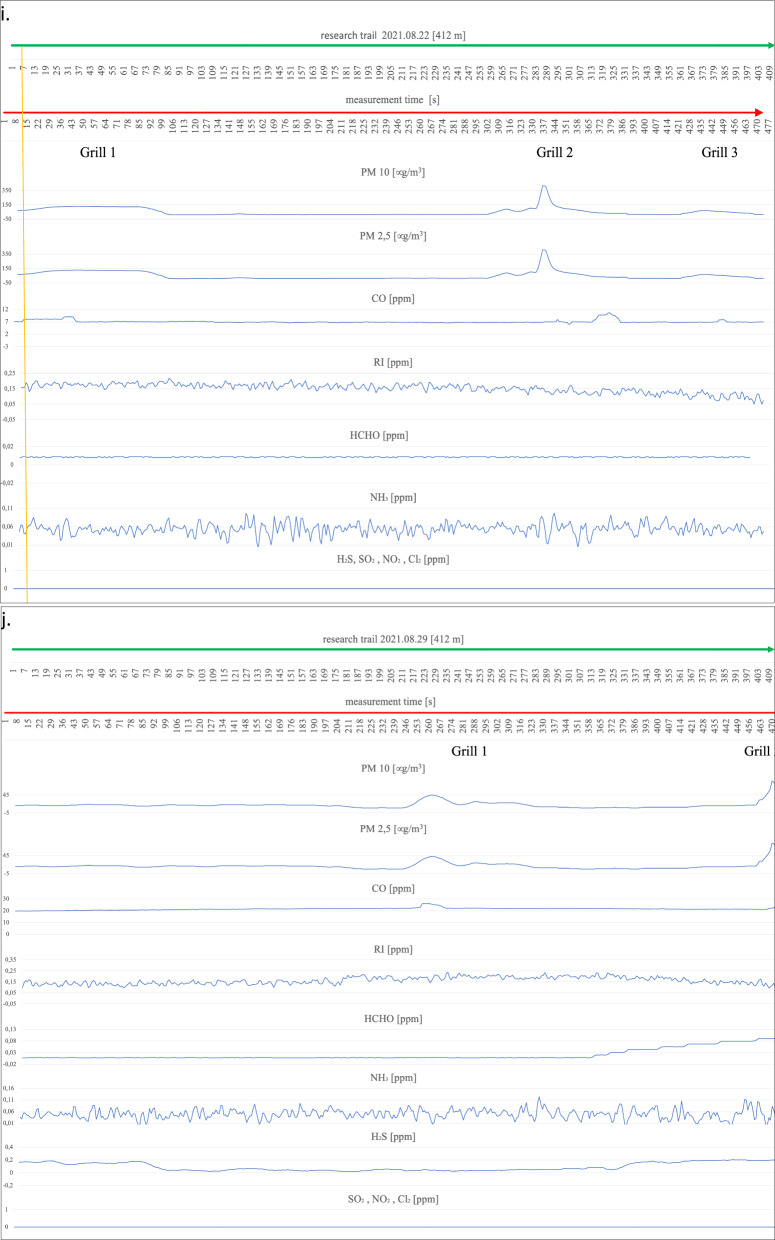

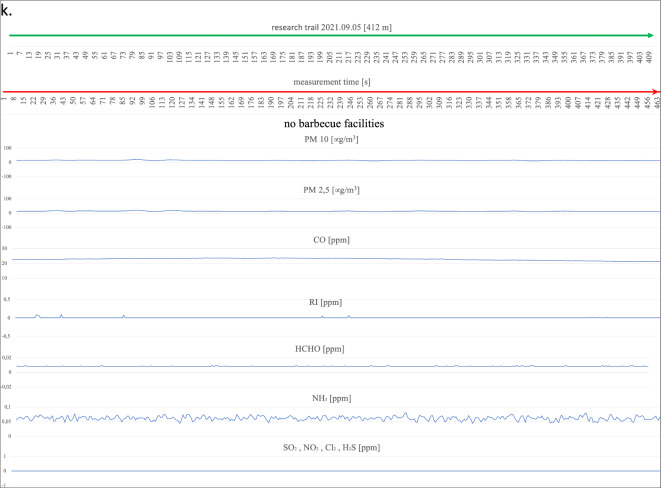
Summary of measurements of gases emitted by active barbecue equipment taken on each test day.

On the first measurement day (Fig. 4a), three working barbecue units were located, and it was noted for barbecue units No. 1 and 3 that the degree of ignition and the advancement of grilling of meat dishes was in the middle phase of operation. Barbecue No. 2 did not have food products placed on it for cooking and was in the quenching phase. The extinguishing hearth (grill no. 2), despite its low gas emissions (min. 22 µg/m^3^, max. 75 µg/m^3^ for PM2.5 and PM10), shows an impact on the air condition in a radius of up to 9 m. An increase in parameter levels can be seen by comparing the average measurement (17–18 µg/m^3^ for PM2.5 and PM10) at locations distant from the grills to the site of the grills and the start of a clear increase in the measured parameters until the point where these parameters drop to background levels (approximately 17 µg/m^3^). A similar spread of emissions is shown by grills 1 and 2 within a 10 m radius of the hearth. Outside the indicated emission range, grills 1 and 2 emit above-normal levels of particulate matter (up to 423 µg/m^3^ for PM10 and 417 µg/m^3^ for PM2.5). CO concentrations also show an increasing trend in the vicinity of the barbecues and a decreasing recorded value when moving away from the barbecue equipment. There is also a noticeable spread of CO over a large area around the grills, taking values from a minimum of 10.095 ppm to a maximum value of 17.951 ppm. The relatively flat level of the graph for CO and its large spread, but with a low nominal value, is probably related to the very weak wind force recorded (3.6 km/h) at the time of the measurements. The irritants (RI) in the vicinity of the barbecues also show a slight increasing trend; in particular, this increase is noticeable at barbecue No. 2, where the RI takes a maximum value of 0.159 ppm. The gases HCHO, NH_3,_ and H_2_S do not show an upward trend during the measurement related to the presence of active barbecue equipment. The other gases, SO_2_, NO_2,_ and Cl_2,_ were not recorded by the measurement systems. On the following measurement day (Fig. 4b), two active grills were recorded during the measurement cycle. We can determine the radius of influence of the first as well as the second of the grills on the basis of the increases and decreases of the measured parameters PM2.5 and PM10, and it is at the level of 14 m. Grill No. 1 shows significantly higher emission values for the individual gases, i.e. for PM10 from 20 µg/m^3^ to 719 µg/m^3^ at the device itself, and for grill No. 2, these values take on PM10 from 20 µg/m^3^ to 231 µg/m^3^. A slight upward trend in the vicinity of the grills also occurs at Grill No. 1 for CO from 15.663 ppm to 17.291 ppm and a marked increase in H_2_S levels starting from Grill 1 through the continuation to Grill 2 at levels ranging from 0.008 ppm to 0.242 ppm. For CO, this increase is undoubtedly related to the quality of the fuel and the direction (WNW) of the wind. The radius of influence of the grills in question at the time of measurement was in the range of 12 m. A survey of emissions from barbecue burners on the eleventh of August revealed three active barbecue devices (Fig. 4c). Grill No. 2 showed the highest emissions (PM10 ranging from 19 µg/m^3^ to 667 µg/m^3^), with the smallest range with a radius of approximately 9 m. Grill Nos. 1 and 2 were characterised by low emissions in the range for PM10 between 18 and 294 µg/m^3^, while values ranging from 23 µg/m^3^ to 108 µg/m^3^ were recorded for Grill No. 3. Increases in readings for CO were also recorded at barbecue No. 3 (17.221 ppm), while the other barbecues (1 and 2) showed no increase peaks for CO, which was probably related to the high wind force (18.7 km/h), which diluted the emissions of this gas. In addition, despite the windy weather, emission peaks were recorded for HCHO (0.04 ppm) and RI (0.007 ppm) and slightly for Cl_2_ (0.112 ppm) in the case of barbecue No. 1. During measurements taken on the eighteenth of August, emissions from three barbecues were also recorded (Fig. 4d). Two of the grills (1 and 2) were in the extinguishing phase, but with fried products on the grates and grill No. 3 was also fully lit with food products laid down, but only in the initial grilling phase. The results obtained from the PM10 and CO measurements were used to determine the radius of influence of the barbecue fumes. Barbecue Nos. 1 and 2 raised the readings of the aforementioned parameters within a radius of approximately 10 m, while barbecue No. 3 affected the air within a radius of 20 m, polluting the air to a significant extent. At barbecue No. 3, readings for PM10 ranged from 4 µg/m^3^ to 904 µg/m^3^ at the unit itself. For undetermined reasons (the wind force was only 5.5 km/h), both the RI (0 ppm–0.203 ppm) and NH_3_ (0.028 ppm–0.055 ppm) measurements had high variability and values compared to the other measurement days. The obtained RI and NH_3_ results, however, cannot be linked to the barbecue equipment. It is likely that the influence on these parameters was external and related to the southern (SW—location of the Jaworzno coal-fired power plant) wind direction. On the next measurement day (Fig. 4e), the emission values from the two grills and their range of influence were measured. Grill No. 1 showed an increase in emissions for H_2_S (0–0.088 ppm) and for PM between 16 µg/m^3^ and 853 µg/m^3^ in the vicinity of the grill. We can determine the range (radius) of the impact of barbecue exhaust from barbecue No. 1 from the increase in PM10 parameters to be approximately 9 m. Barbecue No. 2, in addition to PM10 emissions (17 µg/m^3^–942 µg/m^3^), shows increased emission parameters for CO to 32.076 ppm and H_2_S to 0.016 ppm in the immediate vicinity of the unit. We can read the radius of influence of Grill No. 2 from the graph at 14 m. Measurements carried out on the first of August (Fig. 4f) due to the rainy weather showed the presence of one active grill. Elevated gas emissions were found within 10 m of the unit. High PM10 concentrations of up to 978 µg/m^3^ were recorded, with an average background level of approximately 14 µg/m^3^. In addition, the active grill showed high emissions of CO (5.615 ppm), RI (0.069 ppm) and, with a slight shift in line with the direction of the air masses, HCHO at the highest concentration of 0.044 ppm. On the eighth of August, emissions were recorded from two grills (Fig. 4g). Unit No. 1 covered a circle of approximately 15 m radius with emissions and showed an increase in PM10 emissions of 112 µg/m^3^. Grill No. 2 showed emissions for PM10 ranging from 10 µg/m^3^ to 240 µg/m^3^. The CO concentration that can be associated with the barbecue process extends over a large area of approximately 170 m and does not reach high concentrations, reaching a maximum of 14.916 ppm. The other measured gas concentrations are not related to barbecuing and, as with the measurements on the eighteenth of July, are related to wind direction (WSW) with external industrial pollution. The highest number of active barbecues was recorded on the fifteenth of August (Fig. 4h). Barbecue No. 1 particularly stood out in terms of the extent of its impact on the surrounding air, contributing to a maximum increase in PM10 emissions from 10 µg/m^3^ to 1001 µg/m^3^ over 48 m (24 m radius). The other emitted gases from Grill No. 1 spreading over such a large area were CO reaching a concentration at the grill of 49.599 ppm and RI (0.138 ppm). In addition, an elevated value for HCHO (0.066 ppm) could be seen at the unit itself. Grill No. 2 covers an area with an 8-m radius and has a rather flat emission curve for PM10 at the highest level of 486 µg/m^3^ and for CO 27.717 ppm. However, it was not possible to tell without disturbing beachgoers whether it was in the quenching stage or whether it was being fuelled with high-quality fuel due to the closed design of the unit (furnace cover). It could also not be ruled out that the design of the appliance itself was causing a reduction in the amount of gases coming out of it. During the entire test cycle, all measurements were taken in the vicinity of the uncovered grills (hearths without lids). The radius of influence of the other grills was approximately 9 m for grill 3, approximately 20 m for grill 4, and approximately 14 m for grill 5. Grills 3, 4, and 5 also had similar emission ranges for PM2.5 and PM10 ranging from 40 µg/m^3^ to 970/988 µg/m^3^. Grills 3, 4, and 5 also increased CO in the ambient air to the extent of 33.665 ppm for No. 4, 49.182 ppm for No. 5 and 54.923 ppm for Grill No. 5. There was also an increase in HCHO emissions to 0.02 ppm for grill No. 4. Measurements of grill exhaust concentrations taken on the twenty-second of August (Fig. 4i) showed an increase in concentration ratios for three grills. Grill 1 and 3 were in the extinction phase (with food products on the grill) and emitted PM10 and PM2.5 and CO over a considerable distance (grill 1 within 20 m, grill 3 within 15 m). PM10 reached its highest concentrations at 129 µg/m^3^ for barbecue No. 1 and 68 ppm for barbecue No. 3, and CO at its maximum concentration was recorded at 8.981 ppm for barbecue No. 1 and 7.888 ppm for barbecue No. 3. Barbecue No. 2 also polluted the air with exhaust fumes within a considerable radius of influence (approximately 30 m), which was probably, for all the barbecues on this measurement day, related to the low wind speed (2.8 km/h) and the relatively low atmospheric pressure (978 hPa). In the case of the measurements taken for PM10, the highest concentration was recorded at the device itself at a maximum level of 415 µg/m^3^ and for CO approximately 10 m from the barbecue (10.636 ppm). On the twenty-ninth of August, the impact on the atmospheric air composition of two active grills was recorded (Fig. 4j). Barbecue No. 1 emitted fumes within a radius of approximately 15 m, and the highest concentration for PM reached 45 µg/m^3^ and for CO at a maximum level of 26.211 ppm. No. 1 barbecue had no food products on it, and the fuel itself was in the final burnout stage. Barbecue No. 2, in contrast to barbecue No. 1, was only fired up using kindling, the fuel in the barbecue was charcoal briquettes, and the food to be cooked had not yet been placed on the grate. The radius of influence of barbecue No. 2 (approximately 6 m) on the composition of the surrounding air was relatively small compared to all previous measurements and was probably due to the initial phase of its use. Maximum concentration readings for PM10 were measured at 84 µg/m^3^ and for CO at 22.865 ppm. The last measurements along the designated measurement route on the beach at the "Sosina" reservoir were made on the fifth of September (Fig. 4k). Although it was a cloudless day with no precipitation, it is likely that the temperature at 18 °C effectively limited the number of people using the facility. Additionally, no active barbecues were recorded on this day and at the scheduled measurement time. For PM10, the instruments showed the highest concentration at an average level of 14 µg/m^3^, and for CO, the concentration was recorded at an average level of 22.8 ppm^27^. Reference levels of pollutants in the air for the protection of human health were taken into consideration based on the impact of CO, RI, HCHO, NH_3_, H_2_S, SO_2_, NO_2_, and Cl_2_ for a 30-min inhalation and the average value measured over 24 h for PM10^28–30^. These were compared with the highest recorded values during the measurement for the components mentioned (Table 2).

**Table 2:** Comparison of reference values of concentrations of gases and suspended dust with maximum measured values for combustion emissions from grills.

Medium	Reference values	Maximum measurement	Exceedance of the recommended reference value	Measurement date	Grill no
	ppm	ppm	%	DD/MM/YYYY	No
H_2_S	0.028	0.347	1239	27.06.2021	2
NH_3_	0.57	0.092	–	01.08.2021	1
Cl_2_	0.033	0.112	34	11.07.2021	2
HCHO	0.040	0.066	165	15.08.2021	1
SO_2_	0.19	0	–	–	–
CO	17.7	54.923	310	15.08.2021	5
NO_2_	0.013	0	–	–	–
RI	0.076	0.294	386	25.07.2021	1

Suspended dust	μg/m^3^	μg/m^3^	%	DD/MM/YYYY	No
PM10	45	1001	2224	15.08.2021	1

The highest exceedance of the recommended values was observed for CO at 310% near Grill No. 5 on August 15 and HCHO at 165% near Grill No. 1 on the same day. RI was 386% higher than the reference values near Grill No. 1 on August 25. An exceptionally high result of 1239% for H_2_S was recorded near Grill No. 2 on June 27. Moreover, the Cl_2_ concentration near Grill No. 1 was 34% higher than the safe concentration on July 11. Conversely, NH_3_, SO_2_, and NO_2_ did not surpass the reference values. The highest exceedance of PM10 was recorded at 2224% near Grill No. 1 on August 15 (Table 3).

**Table 3:** A summary of the maximum results for each barbecue of the measurements obtained during the exhaust test.

Measurement date/grill no	Medium/result	Medium/result	Medium/result	Medium/result	Medium/result	Medium/result	Medium/result	Medium/result	Medium/result
27.06.2021	H_2_S	NH_3_	CL_2_	HCHO	SO_2_	CO	NO_2_	RI	PM10
Grill nr 1	0.267	0.048	0	0.009	0	14.422	0	0.093	423
Grill nr 2	0.347	0.054	0	0.009	0	13.803	0	0.159	77
Grill nr 3	0.282	0.049	0	0.008	0	10.503	0	0.174	417
04.07.2021	H_2_S	NH_3_	CL_2_	HCHO	SO_2_	CO	NO_2_	RI	PM10
Grill nr 1	0	0.056	0	0.008	0	16.225	0	0.208	745
Gril nr 2	0.181	0.055	0	0.007	0	15.262	0	0.143	228
11.07.2021	H_2_S	NH_3_	CL_2_	HCHO	SO_2_	CO	NO_2_	RI	PM10
Grill nr 1	0	0.066	0.054	0.024	0	15.134	0	0	297
Grill nr 2	0	0.056	0.112	0.008	0	14.783	0	0.007	667
Grill nr 3	0	0.066	0.048	0.008	0	17.288	0	0	185
18.07.2021	H_2_S	NH_3_	CL_2_	HCHO	SO_2_	CO	NO_2_	RI	PM10
Grill nr 1	0	0.048	0	0.008	0	24.163	0	0.082	416
Grill nr 2	0	0.034	0	0.008	0	23.995	0	0.043	319
Grill nr 3	0	0.050	0	0.009	0	22.932	0	0.153	942
25.07.2021	H_2_S	NH_3_	CL_2_	HCHO	SO_2_	CO	NO_2_	RI	PM10
Grill nr 1	0.088	0.061	0	0.008	0	14.07	0	0.294	865
Grill nr 2	0.007	0.043	0	0.009	0	32.076	0	0.226	942
01.08.2021	H_2_S	NH_3_	CL_2_	HCHO	SO_2_	CO	NO_2_	RI	PM10
Grill nr 1	0	0.092	0	0.009	0	4.484	0	0.067	996
08.08.2021	H_2_S	NH_3_	CL_2_	HCHO	SO_2_	CO	NO_2_	RI	PM10
Grill nr 1	0	0.055	0	0.008	0	13.385	0	0.175	112
Grill nr 2	0	0.042	0	0.009	0	14.499	0	0.178	240
15.08.2021	H_2_S	NH_3_	CL_2_	HCHO	SO_2_	CO	NO_2_	RI	PM10
Grill nr 1	0	0.057	0	0.066	0	49.599	0	0.025	1001
Grill nr 2	0	0.068	0	0.008	0	25.407	0	0.085	486
Grill nr 3	0.198	0.066	0	0.008	0	29.896	0	0.059	973
Grill nr 4	0.275	0.058	0	0.02	0	46.663	0	0.022	998
Grill nr 5	0.217	0.057	0	0.008	0	54.923	0	0.053	976
22.08.2021	H_2_S	NH_3_	CL_2_	HCHO	SO_2_	CO	NO_2_	RI	PM10
Grill nr 1	0	0.059	0	0.009	0	9.033	0	0.177	130
Grill nr 2	0	0.080	0	0.009	0	6.798	0	0.155	415
Grill nr 3	0	0.065	0	0.009	0	7.888	0	0.119	68
29.08.2021	H_2_S	NH_3_	CL_2_	HCHO	SO_2_	CO	NO_2_	RI	PM10
Grill nr 1	0.044	0.076	0	0.008	0	26.211	0	0.113	45
Grill nr 2	0.199	0.083	0	0.021	0	22.133	0	0.103	84
05.09.2021	H_2_S	NH_3_	CL_2_	HCHO	SO_2_	CO	NO_2_	RI	PM10
No grills	0	0.078	0	0.009	0	22.8	0	0	20

**Figure 5:**
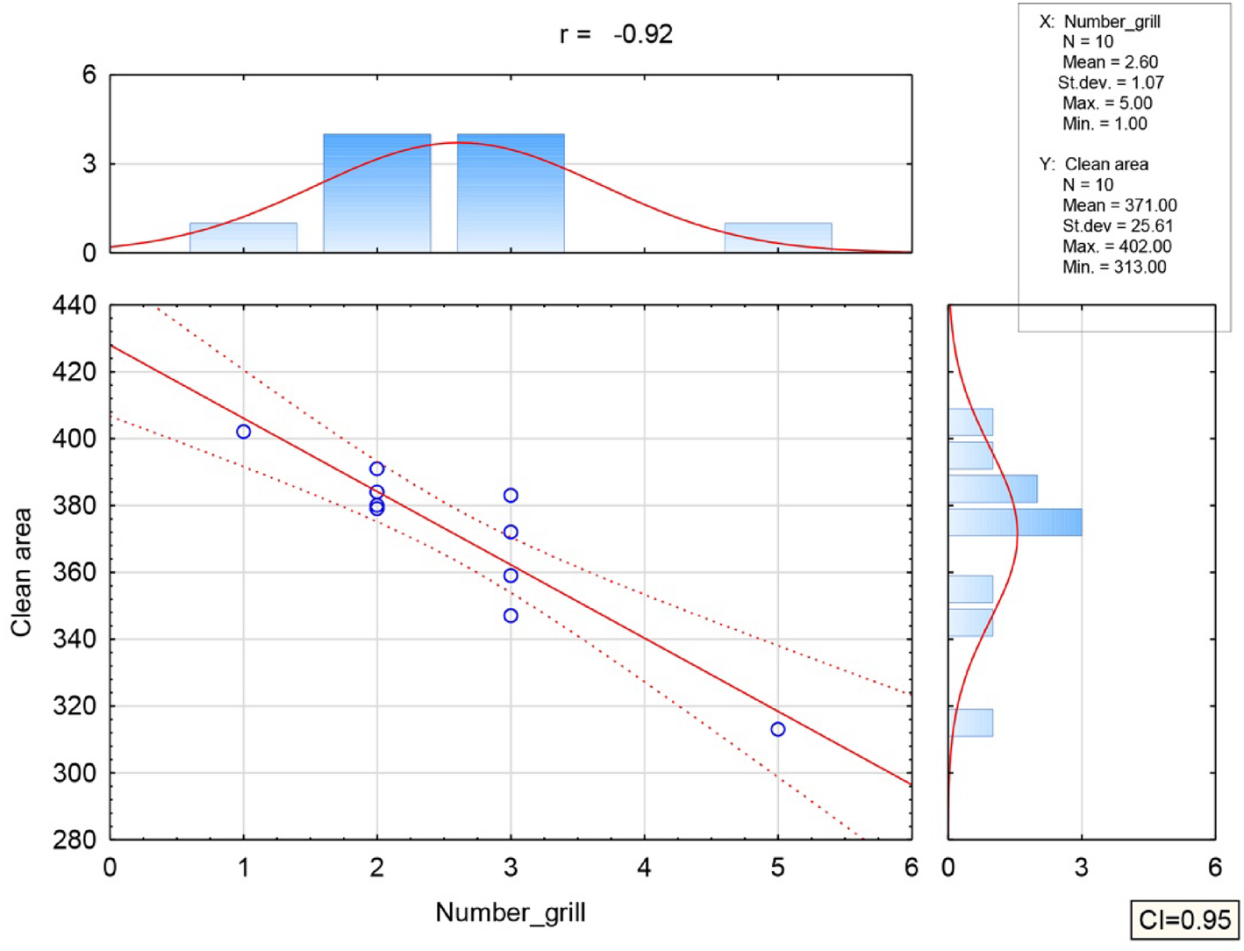
Graphic representation between the number of operating grills and the area affected by exhaust fumes from the grill stands.

Relationships were calculated between the number of barbecues in operation and the area affected by barbecue fumes from barbecue stands. Based on the Karl Pearson correlation coefficient, there was a high correlation between the variables indicating air pollution from barbecue fumes in the measured area as the number of barbecue stands increased (R = − 0.92), Fig. 5.

**Figure 6:**
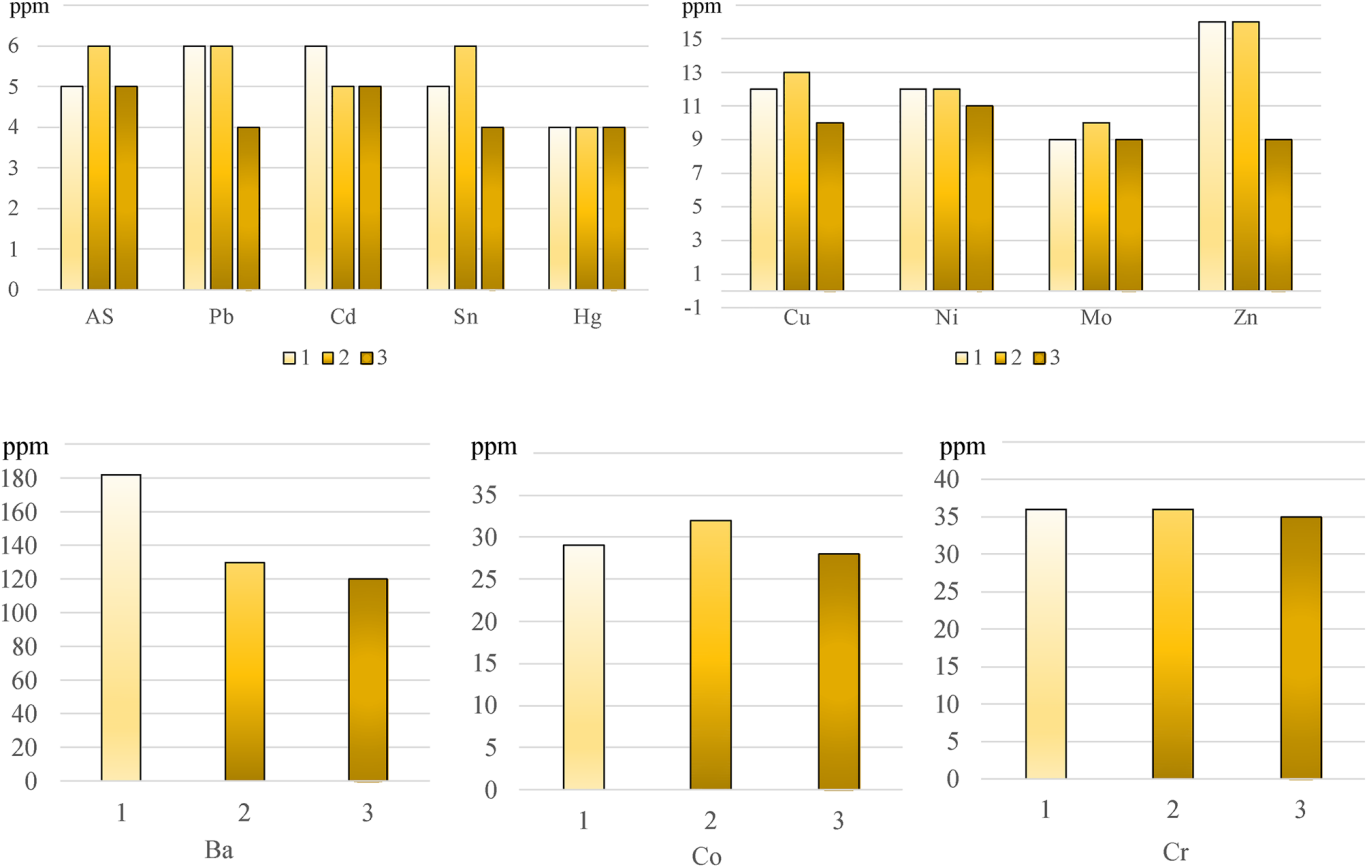
Graphical representation of the distribution of the elemental content of the beach sands across the samples.

As a result of the analysis for the presence of elements in the beach sands located near the grills in use (measurements 1 and 2) and one site away from these appliances (measurement 3), a distribution of the concentration of these elements in the analysed sites was obtained (Fig. 6). Of the twelve elements determined, higher concentrations were found for eight elements (Pb, Sn, Cu, Ni, Zn, Ba, Co, Cr) during measurements near the grills. Measurements taken at a location away from the barbecue equipment showed lower concentrations of all the elements determined.

Table 4 shows the reference values (under standard) for the content of individual elements in Group I soils—sports and recreation areas. Apart from cadmium (Cd), whose reference value is exceeded almost twice, the other elements do not show concentrations above the recommended values. The measured values in the beach sands for As, Cr, Zn, Cu, Mo, and Pb can be described as very low in relation to the limit values. However, a slight increasing trend is noticeable for the concentrations of these elements in the sites (1 and 2) directly used for food processing in barbecue equipment. For nickel (Ni), there is a concentration of 12 ppm, which is close to the limit (15 ppm). Similarly, measurements of mercury (Hg) in beach sands show concentrations of this metal less than 1 ppm from the limit (5 ppm). In the case of mercury, there was no difference between its content in the beach sands located near the barbecues and the measurement taken at a distance from the barbecue sites.

**Table 4:** Summary of concentrations of selected elements in beach sands.

Sample No	As	Ba	Cr	Sn	Zn	Cd	Co	Cu	Mo	Ni	Pb	Hg
	ppm	ppm	ppm	ppm	ppm	ppm	ppm	ppm	ppm	ppm	ppm	ppm
1	5	182	36	5	16	6	29	12	9	12	6	4
2	6	130	36	6	16	5	32	13	10	12	6	4
3	5	120	35	4	9	5	28	10	9	11	4	4
Standard	25	400	200	20	500	2	50	200	50	15	200	5

Petrographic analysis performed on the sampled beach sands for the presence of solid contaminants showed differences in both the quality and volumetric quantity of the various elements in the studied material (Table 5).

**Table 5:** Summary of identified solid elements in beach sands.

Solid component	Sample 1	Sample 2	Sample 3
	%	%	%
Rust	0.2	0	0
Metals	0.4	0.1	0.1
Plastics	0.6	1	0.8
Polymers	0.8	1.6	1
Charcoal	0.5	0.8	0
Biomass	1.1	1.1	1.2
Slags	0.1	0.6	0
Fossil coals	0	0.1	0
Glass	0.3	0.2	0.2
Mineral matter (sand)	96	94.5	96.7
Total	100	100	100

**Figure 7:**
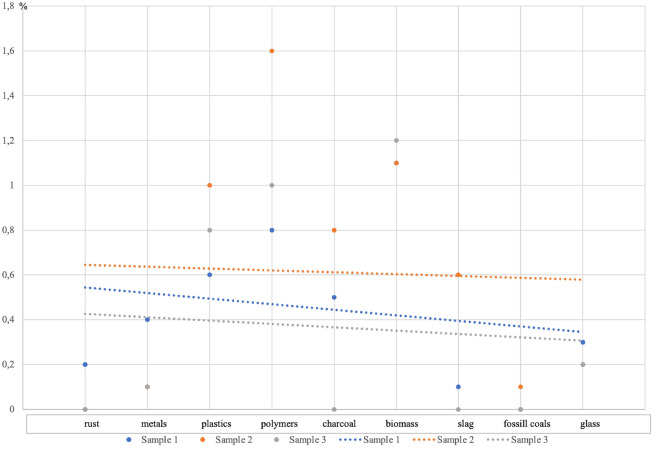
Trend lines of solid contaminants in beach sands obtained from petrographic analysis.

The linear regression performed for the data obtained from the petrographic analysis (Table 5) confirms the assumption that the farther the site is from the barbecue sites, the lower the concentration of anthropogenic pollutants (Fig. 7).

**Figure 8:**
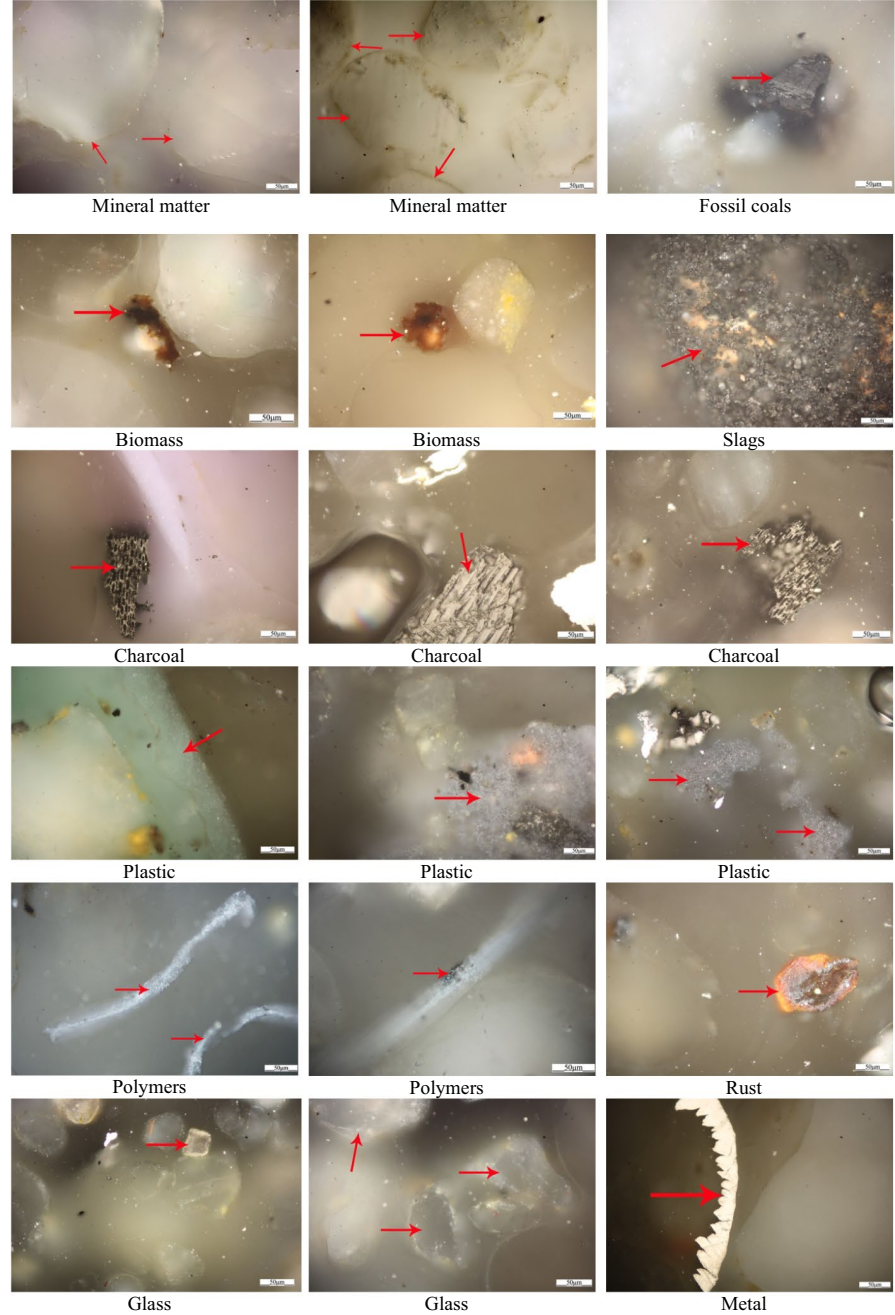
Microscopic images of solid constituents present in beach sands.

Grains of quartz were the main constituent of the material studied; in addition to this building block (sand), fragments of rust, metals, plastics, polymers, charcoal, biomass, slags, fossil coals and glass were also identified (Fig. 8). The remaining mineral components were not identified separately but together with quartz were generally classified as mineral matter^31,32^. The least amount of solid elements apart from sand grains was found in sample 3 located approximately 30 m away from the barbecue pit. Charcoal and fossil coals and slags (contaminants associated with the use of the hearths) were not found in the sample. However, the content of polymers, fibrous material from cigarette filters^33^, plastics (packaging), metal (caps) and glass is indicative of the use of this area by beachgoers for both sunbathing and communication.

**Figure 9:**
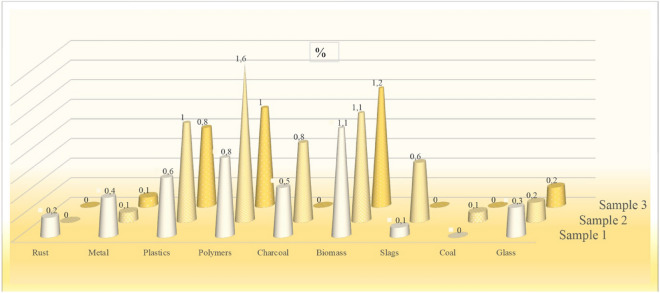
Percentage of individual solids in the beach sand samples studied.

In samples 1 and 2, taken at the site of the barbecue hearths, many more solid elements were isolated than in sample 3, including charcoal and slag fragments, which are closely related to the thermal processing of food using barbecue equipment. In addition, there are fragments of metal, plastic, glass and rust associated with the consumption of barbecue food in the immediate vicinity of beverages and food packaging^34,35^. In addition, thermal charcoal was identified in sample 2, probably originating from charcoal briquettes contaminated with this fuel. A similar content of nonthermally processed biomass was found in all three samples, the biomass most likely originating from the numerous trees and scrub located in the bathing area^36,37^ (Fig. 9).

## Summary

The use of the beach and the preparation of food by means of heat treatment of food products using barbecue equipment result in the generation of contaminants both in the beach sands and in the air. Analysis of the sands did not reveal, apart from cadmium (Cd), high concentrations of carcinogenic elements when measurements were taken near the barbecues and at a distance from the barbecues. However, a difference was found between the element content of sands located directly next to the barbecue sites (higher element concentration) and sands further away from these sites. Similar differences between the contamination of beach sands were shown by testing for solid elements such as rust, metals, plastics, polymers, charcoal, biomass, slags, fossil coals, and glass. Immediately adjacent to the grills, the accumulation of solid contaminants clearly increased up to 0.6% in the case of polymers and slags. The greatest adverse impact on the comfort and health of people using beaches located next to leisure centres was found in the case of air pollution from exhaust fumes emitted from barbecues. PM10 and PM2.5 particles contained in the fumes emitted by the barbecues often exceeded the permissible concentrations not only at the devices themselves but even within a radius of several metres from them. Increased concentrations within a radius of a few metres from the grills were also recorded for measurements taken for CO, RI, HCHO, and NH_3_. It should be emphasised that in the case of this type of exposure to fumes within a radius of a few to several metres from the emitter, not only the users of the grills themselves but also other members of the public using recreational facilities are exposed to their inhalation.

## Data Availability

The datasets used and/or analysed during the current study available from the corresponding author on reasonable request.
